# *Notes from the Field:* Epidemiologic Characteristics of SARS-CoV-2 Recombinant Variant XBB.1.5 — New York City, November 1, 2022–January 4, 2023

**DOI:** 10.15585/mmwr.mm7208a4

**Published:** 2023-02-24

**Authors:** Elizabeth Luoma, Rebecca Rohrer, Hilary Parton, Scott Hughes, Enoma Omoregie, Faten Taki, Jade C. Wang, Saymon Akther, Helly Amin, Carolyn Chang, Iris Cheng, Steve Di Lonardo, Meredith Eddy, Lauren Firestein, Wenhui Li, Michelle Su, Ellen H. Lee

**Affiliations:** 1 New York City Department of Health and Mental Hygiene, Long Island City, New York.

The SARS-CoV-2 Omicron XBB.1.5 variant, a recombinant variant of Omicron BA.2.75 and BA.2.10, was first detected in New York City (NYC) in October 2022. As of January 7, 2023, XBB.1.5 was the predominant variant in NYC, accounting for 81% of sequenced specimens; at that time, only 26% of sequenced specimens nationwide were XBB.1.5 (*1*). In addition, in December 2022, only 5% of sequenced genomes in the rest of New York were XBB.1.5, suggesting that NYC was likely the epicenter of XBB.1.5’s emergence in the United States (*2*). The World Health Organization has noted that XBB.1.5 does not carry any mutation known to be associated with a potential change in severity, such as the Delta spike mutation P681R; however, there are currently limited data available about disease severity in human populations (*3*). Because NYC witnessed the emergence of XBB.1.5 before much of the United States, and the NYC Department of Health and Mental Hygiene (DOHMH) routinely links whole genome sequencing and epidemiologic data, DOHMH is uniquely positioned to characterize this subvariant. Although a higher percentage of patients infected with XBB.1.5, compared with those infected with a co-circulating variant, were younger, identified as racial and ethnic minorities, and lived in high-poverty neighborhoods, and a lower percentage had completed a primary COVID-19 vaccination series with ≥1 dose of monovalent vaccine booster, there was no evidence of a difference in disease severity.

SARS-CoV-2 specimens collected from NYC residents at five DOHMH COVID-19 Express laboratories, 190 outpatient clinics, and 11 emergency departments across all boroughs within the NYC municipal hospital system were sequenced at DOHMH’s Public Health Laboratory or the Pandemic Response Laboratory, which has operated in Manhattan since September 2020. Sequenced isolates were matched to the DOHMH COVID-19 surveillance database (Maven, version 5.5.1; Consilience Software), Citywide Immunization Registry, health information exchanges, and e-Vitals Death Registry to identify demographic characteristics and previous SARS-CoV-2–positive test results, monovalent immunization history,* hospitalization status, and vital status, respectively. Persons infected with XBB.1.5 (3,019) were compared with persons infected with BQ.1^†^ (6,067) during November 1, 2022–January 4, 2023, because both variants were co-circulating in NYC starting in November 2022, and BQ.1 was the predominant variant in NYC when XBB.1.5 emerged. Comparisons across categorical characteristics were made using Pearson chi-square or Fisher’s exact test; continuous variables were compared using the Kruskal-Wallis test. Analyses were performed using SAS statistical software (SAS Enterprise Guide; version 7.1; SAS Institute). This activity was reviewed by the NYC DOHMH Institutional Review Board and was determined to be public health surveillance and therefore not subject to human subjects review.

During November–December 2022, the percentage of sequenced SARS-CoV-2 isolates in NYC identified as XBB.1.5 increased eightfold, from 8% to 72%. Compared with patients infected with BQ.1 (p<0.001), those with XBB.1.5 infections tended to be younger (median age = 41 years [XBB.1.5] versus 44 years [BQ.1]), Hispanic or Latino or non-Hispanic Black or African American (Black) (68.1% versus 61.5%), and residents of the Bronx, Brooklyn, or Queens (82.6% versus 76.1%); a higher percentage lived in high- or very high–poverty neighborhoods (43.2% versus 41.9%) (Table). The percentage who had received a primary COVID-19 vaccination series and ≥1 dose of monovalent vaccine booster was lower among patients with XBB.1.5 infections (41.1%) than among those with BQ.1 infections (46.0%). The percentages of XBB.1.5 and BQ.1 patients whose specimen was collected ≥90 days after a previous collection of a specimen with a SARS-CoV-2–positive test result, which could suggest possible reinfection, were similar (25.2% [XBB.1.5]; 25.4% [BQ.1]). No difference in the proportion of patients hospitalized or those who died was observed, suggesting no significant difference in disease severity.

**TABLE:** Characteristics of persons infected with SARS-CoV-2 XBB.1.5 and BQ.1* variants^†^ — New York City, November 1, 2022–January 4, 2023

Characteristic	SARS-CoV-2 Omicron variant no. (column %)		p-value^§^ (XBB.1.5 versus BQ.1)
	XBB.1.5 (n = 3,019)	BQ.1 (n = 6,067)	
**Median age, yrs (IQR)**	41 (27–57)	44 (30–59)	<0.001
**Age group, yrs^¶^**			
0–17	340 (11.3)	497 (8.2)	<0.001
18–44	1,362 (45.1)	2,593 (42.7)	
45–64	897 (29.7)	1,903 (31.4)	
65–74	252 (8.4)	678 (11.2)	
≥75	166 (5.5)	396 (6.5)	
**Sex^¶^**			
Female	1,767 (58.5)	3,573 (58.9)	0.72
Male	1,252 (41.5)	2,491 (41.1)	
Race and ethnicity^¶,^**			
Asian or Pacific Islander	302 (11.4)	715 (13.4)	<0.001
Black or African American	791 (29.9)	1,479 (27.7)	
Hispanic or Latino	1,012 (38.2)	1,802 (33.8)	
White	519 (19.6)	1,277 (24.0)	
Other	23 (0.9)	57 (1.1)	
**Borough of residence^¶^**			
The Bronx	562 (18.7)	993 (16.4)	<0.001
Brooklyn	1,103 (36.7)	2,097 (34.7)	
Manhattan	454 (15.1)	1,188 (19.6)	
Queens	819 (27.2)	1,511 (25.0)	
Staten Island	71 (2.3)	257 (4.3)	
**Neighborhood poverty level,^¶,††^ (% of persons)**			
Low (<10)	329 (11.5)	767 (13.3)	<0.001
Medium (10–19.9)	1,300 (45.3)	2,591 (44.8)	
High (20–29.9)	745 (26.0)	1,604 (27.7)	
Very high (≥30)	495 (17.2)	824 (14.2)	
**Monovalent vaccination history** ^§§^			
No recorded dose	746 (24.7)	1,386 (22.9)	<0.001
Partially immunized	159 (5.3)	287 (4.7)	
Primary series only	873 (28.9)	1,602 (26.4)	
Primary series and monovalent booster vaccine dose	1,241 (41.1)	2,792 (46.0)	
**Outcomes**			
**Repeat positive test result** ^¶¶^			
Yes	762 (25.2)	1,543 (25.4)	0.84
No	2,257 (74.8)	4,524 (74.6)	
COVID-19 hospitalization***			
Yes	219 (7.3)	389 (6.4)	0.13
No	2,800 (92.7)	5,678 (93.6)	
**COVID-19 death** ^†††^			
Yes	24 (0.8)	38 (0.6)	0.36
No	2,995 (99.2)	6,029 (99.4)	

* BQ.1 included all descendant lineages of BQ.1 (e.g., BQ.1.1 and BQ.1.x).

^†^ Classified by Pangolin identification of lineage. https://pangolin.cog-uk.io/

^§^ p-values from Pearson chi-square test, Fisher’s exact test, or Kruskal-Wallis test as indicated, comparing persons with XBB.1.5 sequences and BQ.1 sequences.

^¶^ Denominators represent persons with known age, sex, race and ethnicity, borough of residence, and neighborhood poverty level; age was missing for two persons infected with XBB.1.5 variant; sex was missing for three persons infected with BQ.1 variant; race and ethnicity was missing for 1,109 persons, including 372 infected with XBB.1.5 variant and 737 infected with BQ.1 variant; borough of residence was missing for 31 persons, including 10 infected with XBB.1.5 variant and 21 infected with BQ.1 variant; and neighborhood poverty level was missing for 431 persons, including 150 infected with XBB.1.5 variant and 281 infected with BQ.1 variant.

** All persons who identified as Hispanic or Latino (Hispanic), regardless of race, are classified as Hispanic; all other race and ethnicity categories are non-Hispanic.

^††^ Neighborhood poverty level was defined as the percentage of residents in a zip code tabulation area with household incomes of <100% of the federal poverty level, per the American Community Survey 2014–2018.

^§§^ Monovalent vaccination history was categorized into four groups of monovalent vaccine doses received ≥14 days before diagnosis: 1) no recorded dose (zero doses), 2) partially immunized (≥1 dose of an mRNA vaccine), 3) primary series only (≥2 doses of an mRNA vaccine or 1 dose of a viral vector vaccine), and 4) primary series plus ≥1 dose of an mRNA or viral vector monovalent vaccine booster. Bivalent vaccine booster data had not been matched to the New York City Department of Health and Mental Hygiene’s COVID-19 surveillance database at the time of this analysis.

^¶¶^ A repeat positive test result was defined as a sequenced SARS-CoV-2 isolate collected ≥90 days after collection of a specimen with a SARS-CoV-2–positive antigen or nucleic acid amplification test result. At-home tests were not recorded in surveillance activities.

*** A COVID-19 hospitalization was defined as 1) a confirmed or probable COVID-19 diagnosis 14 days before through 3 days after date of hospital admission, 2) a COVID-19–related hospitalization reported on the death certificate, or 3) a COVID-19–related hospitalization reported from a case investigator.

^†††^ A COVID-19 death was defined as 1) a SARS-CoV-2–positive test result within 30 days of death or 2) a diagnosis of COVID-19 listed on the death certificate as a primary or contributing cause of death.

Limitations of these data are that patients with sequencing results accounted for 4%–12% of laboratory-confirmed SARS-CoV-2 cases diagnosed in NYC in November and December 2022 (*4*); therefore, characteristics of persons with and without sequencing results might differ. Although a higher percentage of patients with sequencing results, compared with those without sequencing results, were aged 18–64 years (74% versus 68%), resided in high- or very high–poverty neighborhoods (42% versus 37%) and in Brooklyn (35% versus 29%), identified as Black (28% versus 20%), and had a COVID-19 hospitalization (7% versus 6%), the percentage with COVID-19 deaths was the same (1%) among all patients with laboratory-confirmed cases, irrespective of sequencing status.

XBB.1.5 emerged rapidly in NYC during November–December 2022 and earlier than in the rest of the United States. Preliminary findings from a sample of sequenced isolates in NYC do not suggest more severe disease among patients infected with XBB.1.5 compared with patients infected with BQ.1; however, these findings might change as more data on these outcomes accumulate. Although a small proportion of laboratory-confirmed SARS-CoV-2 cases in NYC are sequenced, linked epidemiologic and genomic data provide a means to evaluate characteristics of emerging variants, including disease severity, that are important for rapid risk assessment (*3*). Routine linkage of epidemiologic and sequencing data allows tracking of emerging variants and ongoing assessment of reinfection, infection after vaccination, and disease severity.

## Footnotes

* Bivalent booster vaccination data were not available at the time of this analysis because they had not yet been matched to DOHMH’s COVID-19 surveillance database.

† BQ.1 included all descendant lineages of BQ.1 (e.g., BQ.1.1 and BQ.1.x).
